# Roadmap for the management of heart failure patients during the vulnerable phase after heart failure hospitalizations: how to implement excellence in clinical practice

**DOI:** 10.2459/JCM.0000000000001221

**Published:** 2021-12-22

**Authors:** Giuseppe M.C. Rosano, Cristiana Vitale, Marianna Adamo, Marco Metra

**Affiliations:** a Department of Medical Sciences, Centre for Clinical and Basic Research, IRCCS San Raffaele Pisana, Rome; b Cardiology, Department of Medical and Surgical Specialties, Radiological Sciences, and Public Health, University of Brescia, Bresica, Italy

**Keywords:** heart failure, hospitalization, vulnerable phase

## Abstract

Patients discharged after an episode of acute heart failure have an increased risk of hospitalizations and deaths within the subsequent 3 months. This phase is commonly called the ‘vulnerable period’ and it represents a window of opportunity of intervention in order to improve longer term outcomes. Prompt identification of signs of residual haemodynamic congestion is a priority in planning for the out-of-hospital management strategies. Patients will also need to be screened for frailty and have a prioritization of the management of their comorbidities. Life-saving medications should be started together or in a short time and up-titrated (when needed) according to blood pressure, heart rate and concomitant comorbidities. Ideally, patients should be assessed by their general practitioner within 1 week of discharge and have a hospital/clinic follow-up within 4 weeks of discharge. Patients should progressively resume physical activities and adhere to an educational programme with appropriate lifestyle adjustments best implemented during a cardiac rehabilitation programme.

## Introduction

Heart failure is one of the major causes of hospitalizations, death and healthcare costs worldwide.^1–4^

Despite recent progress in the therapy of chronic heart failure therapy, patients discharged after an episode of decompensation are still at high risk for mortality, hospitalization and readmission within the subsequent 3 months. Indeed, approximately 30% of patients hospitalized with heart failure are readmitted within 3 months of discharge, and mortality during this period can approach 10%.^5–8^

**Fig. 1:**
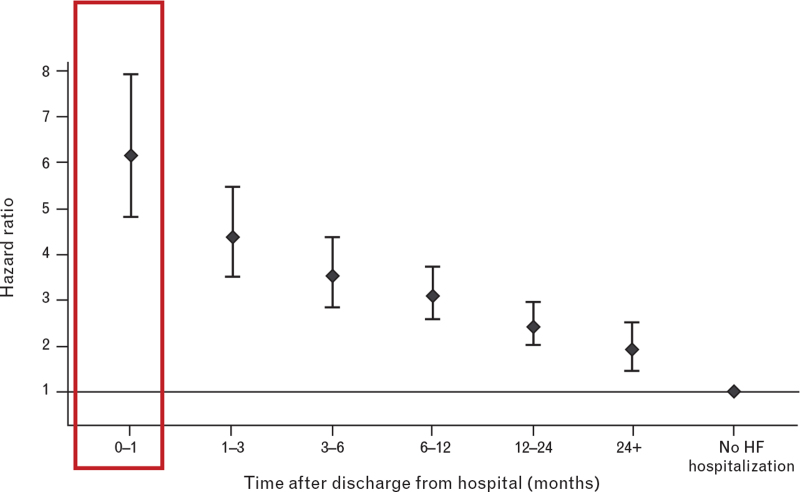
Mortality in the postdischarge period after an episode of acute de-compensation. Adapted with permission from Mebazaa *et al.*^13^ Hazard ratio of all-cause mortality after discharge from hospital for first hospitalization.

Although some studies suggested a higher prevalence of rehospitalizations in patients with heart failure with reduced ejection fraction (HFrEF) than those with preserved ejection fraction (HFpEF), the readmission trends in the vulnerable period seem similar to a larger role of comorbidities in HFpEF.^9–11^ Fewer than half of these rehospitalizations are because of noncardiac causes and two-thirds of the readmissions occurring within 30 days are for nonheart failure primary issues^12^ (Fig. 1).

Given the high risk of poor outcomes, the first 3 months after hospital discharge are commonly defined as the ‘vulnerable period’ or ‘vulnerable phase’.^13–15^ This period starts from discharge after an acute heart failure event, continues through a peridischarge period and lasts for up to 3 months after discharge. The vulnerable period can be divided into three phases: a very early phase (the first month after hospital discharge), an early phase (up to 60 days after hospital discharge), and a late phase (60--90 days from discharge). Due to the risk of early re-admission and poor prognosis, the vulnerable period represents a window of opportunity to identify phenotypes of patients at risk and interventions that may improve prognosis.

A key factor in patient management during the vulnerable phase is the coordination of care between the hospital, the heart failure care team and all the ‘actors’ involved in the delivery of out-of-hospital care. This coordination differs according to different healthcare models in different countries, and it is difficult to find a unifying model that may fit all but certainly fragmentation of care is associated with poor access to follow-up programmes.^12,13^ However, an early visit by the general practitioner (ideally within 1 week after discharge) and short-term specialistic follow-up hospital visits seem to be key factors in improving patient care. An important focus should be given to education programmes for patients and caregivers, in either the hospital or home setting, together with early implementation of cardiac rehabilitation.

Subclinical haemodynamic abnormalities, such as fluid retention or elevated filling pressures occur prior to the occurrence of clear clinically recognized symptoms of congestion in outpatients, thus highlighting the needs for a short-term and long-term care plan to be implemented after discharge to be able to detect in a timely manner early signs of congestion.^16,17^

However, although clinical parameters and biomarkers have been identified for risk stratification in the early posthospitalization period, a reliable model or algorithm applicable to identify those patients at high risk for hospital readmission who may benefit from closer monitoring is still lacking.^18^

## Very early vulnerability phase

The very early phase is associated with a very high risk of rehospitalizations and death, which is often because of the fact that patients are discharged while still haemodynamically unstable and have not been adequately started on life-saving medications with adequate plans for their up-titration. The Guidelines of the European Society of Cardiology (ESC)/Heart Failure Association (HFA) clearly state that patients are eligible for discharge only if they are on evidence-based oral medication, euvolemic and have been haemodynamically stable for at least 24–48 h, with stable renal function for at least 24 h before discharge.^19^ However, this is not always the case and the ACC/AHA registry data reveal that 20% of patients in the United States of America (USA) are discharged despite persistent signs and symptoms of heart failure, including minimal decrease or even increase in body weight.^20–22^

The very early events occurring in the vulnerable period can be expected in patients discharged before complete relief of congestion and it is often associated with the pressure to discharge patients early.^22^ Indeed, the risk of future events in the vulnerable period seems closely related to the length of hospitalization during the acute event and the rate of readmission is higher in countries such as the USA where physicians are encouraged to discharge patients as early as possible (average of 4–5 days) and less common in other countries, such as Japan, where the length of hospitalization is longer. In Europe, the length of hospitalization may vary across countries but it ranges between 8 and 10 days in most European countries.^21,22^ An inverse relationship between length of stay and 30 days readmission rates across different studies has also been shown.^23^

Worsening orthopnoea, low SBP, higher heart rate, low serum sodium, decreased renal function, increased levels of neurohormones, such as antidiuretic hormone and aldosterone, and lower albumin levels are the most common variables that have been associated with an increased risk of hospitalization and death.^18^ These variables have been proposed for risk stratification in the early posthospitalization period.

The assessment of biomarkers, such as natriuretic peptides and cardiac troponins before discharge have been also proposed as a tool to assess the haemodynamic stability and to draft an individualized care management plan. However, the routine measurement of these biomarkers at discharge is not recommended, as no thresholds have been identified to discriminate between patients at low or high risk and their changes seem more related to mortality or long-term risk of readmission. Moreover, a strategy of natriuretic peptides-guided therapy has not been more effective than usual treatment to reduce deaths and hospitalizations in patients with heart failure and either a recent hospitalization or hospitalized at the time of enrolment.^24–27^

The occurrence of a stressor (acute/chronic, internal/external), such as an acute infection, worsening renal function, uncontrolled arrhythmia, and lack of compliance to medications are also potential causes that may alter the labile equilibrium of heart failure patients, thus leading to a decompensation and negative outcomes.^28,29^ Heart failure patients are medically complex, with multiple comorbidities and polytherapy. Around 45% of patients discharged after an acute event are frail.^30^ As frailty is a multidimensional dynamic state, independent of age that makes the individual with heart failure more vulnerable to the effect of stressors, it is important to assess frailty at discharge. In addition, up-titration or even continuation of neurohormonal antagonists may be challenging in these more fragile heart failure patients,^29,31^ given their haemodynamic, renal or tolerability constraints.

Therefore, an integrated care plan focussed on the patient health burden and the prompt implementation of out-of-hospital management strategies are crucial to improve patient care in this phase. The out-of-hospital management should be planned before discharge and an individualized educational programme about self-care should be implemented^18^ in order to promptly identify symptoms or signs of haemodynamic congestion, evaluate the recovery of functional capacity and promote clinical stabilization, to manage the possible comorbidities, thus reducing the risk of complications and decompensation, and progressively implement medical therapy to reach the evidence-based target dosages.^32^ Patients in this phase might still require relatively higher doses of diuretics and vasoactive medications.^33^

Patients and caregiver education and their involvement in symptom monitoring and flexible diuretic dosing play a key role in the subsequent months after discharge, and in particular, in the very early period during which patients can still have subclinical congestion.

Often, because of the haemodynamic instability and/or renal impairment, life-saving medications like beta blockers, angiotensin-converting-enzyme inhibitors (ACE-I) and mineralocorticoid receptor antagonist (MRAs) are discontinued or reduced in the early phases of hospitalization and they are not fully re-instated before discharge. ACE-i/angiotensin receptor blockers (ARBs), beta blockers and MRAs should be started before discharge in all patients with heart failure once congestion has been managed to reduce the risk of heart failure hospitalization and increase survival.^18,34,35^

**Fig. 2:**
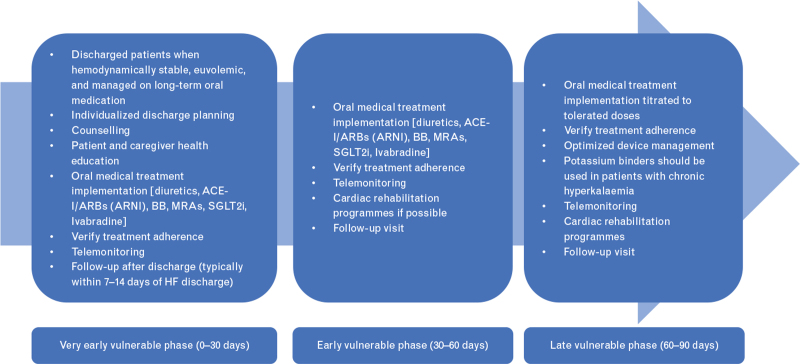
Transition of care during the vulnerable period after hospital discharge. ACE, angiotensin-converting enzyme inhibitors; BB, beta blockers; HF, heart failure; MRA, mineralcorticoid receptor antagonist; RAASi, renin—angiotensin--aldosterone system inhibitors; SGLT2i, sodium-glucose co-transporter2 inhibitor.

However, the implementation of these therapies after discharge should be directed by patient phenotype and spending function on heart rate, blood pressure and renal function/electrolytes. Sodium glucose cotransporter 2 inhibitors (SGLT2i) should be implemented early after discharge as they have a minimal effect on blood pressure while caution may be taken in the initiation and up-titration of an angiotensin receptor-neprilysin inhibitor (ARNI) as it may be less well tolerated because of its hypotensive effects^36–38^ (Fig. 2). Vericiguat and omecamptiv mercabil, once approved by regulatory agencies, could be also started before discharge. However, although omecamtiv mercabil has a neutral effect on heart rate and blood pressure and could be easily and safely started prior to discharge or immediately post hospitalization, vericiguat has peripheral vasodilatatory effects and may be difficult to manage in patients with borderline--low blood pressure. In addition, it did not reduce cardiovascular death or heart failure hospitalizations in patients with high N-terminal pro-B-type natriuretic peptide (NT-proBNP) levels (≥ 8000 pg/ml) in VICTORIA (A Study of Vericiguat in Participants With Heart Failure With Reduced Ejection Fraction) whereas omecamtiv mecarbil was associated with better outcomes in patients with more severe cardiac dysfunction, as shown by a left ventricular ejection fraction (LVEF) of 28% or less (median values).^39,40^

## Early vulnerability phase

The early vulnerable phase lasts for up to 60 days after hospital discharge. The OPTIMIZE-heart failure (Organized Program to Initiate Lifesaving Treatment in Hospitalized Patients With Heart Failure) registry demonstrated a 30% rate of rehospitalization at 60--90 days post discharge and that approximately half of the rehospitalizations were not related to heart failure.^41^

In this period, patients should be adequately de-congested and euvolemic, life-saving medications should be up-titrated wherever possible and patients should undergo a cardiac rehabilitation programme in order to improve symptoms, functional capacity, and quality of life and to prevent rehospitalizations. Given its prognostic benefit, SGLT2i should be implemented in all patients.

According to clinical trials, patients who were on an adequate dose of ACEi at the time of hospitalization can be switched to Sacubitril/Valsartan but patients who were receiving them at a sub-optimal dose should have the ACEi up-titrated before considering the switch^42–44^ (Fig. 2).

Special care should be paid to the speed of up-titration of beta blockers as a fast up-titration may lead to an increased risk of side effects and hospitalizations.^45^ Co-administration of ivabradine and beta blockers is well tolerated and may be more effective than beta blockers alone in reducing the heart rate, improving left ventricular function and exercise capacity.^46^ MRAs should be up-titrated according to blood pressure and K^+^ levels. In patients with hyperkalaemia, the new K-binders (ZS-9 and patiromer) may help to enable the up-titration of RAASi therapy. Iron deficiency should be checked at regular (6-month) intervals and iron-deficient patients should receive ferric carboxymaltose.^47–49^ Oral iron is not recommended as it is not effective in patients with heart failure.

## Late vulnerability phase

The main management aim of this period is to implement guideline-recommended medical and device therapy in order to stabilize the clinical status and to improve the long-term outcome. In this phase, treatment with MRAs and SGLT2i should be implemented in all patients and ARNI started in those patients with a LVEF of less than 35% who are still symptomatic despite guideline-directed medical therapy. ARNI should be given as a replacement for ACEi or ARB when patients who are on these agents are still symptomatic and have raised BNP/NT-proBNP levels. They have been also given to patients not on ACEi/ARBs in recent studies.^45,50^

Although none of these studies was aimed at the assessment of the effects on outcomes, the results showed the safety of this approach. Diuretics should be adjusted in order to maintain euvolemia, and potassium binders should be used to control K levels in those patients with recurrent hyperkalaemia.

In this phase, up-titration to target dosages of life-saving medications should be obtained. However, several data showed that although the rate of prescription of the medications recommended by international guidelines has improved, up-titration of these drugs remains still suboptimal in clinical practice. Fewer than 25–30% of patients receive the target recommended dose of beta blockers and fewer than 50–60% receive target doses of ACEi/ARB in clinical practice.^49,51–55^ Not only therapeutic inertia but also biological limitations, namely hypotension, hyperkalaemia and renal dysfunction, remain major barriers to the implementation and adherence to treatment with agents acting on neurohormonal mechanisms^56–58^ (Fig. 2).

Patients should progressively resume physical activities and adhere to an educational programme with appropriate lifestyle adjustments best implemented during a cardiac rehabilitation programme.^18^

According to a recent Cochrane Systematic Review, Meta-Analysis, and Trial Sequential Analysis the clinical and health-related quality of life benefits of exercise-based cardiac rehabilitation appear to be consistent across trial settings (i.e. centre-based compared with home-based exercise or cardiac rehabilitation), type of rehabilitation (i.e. comprehensive compared with exercise-only exercise-based cardiac rehabilitation programme) and dose of exercise-based cardiac rehabilitation.^59–61^

Rehabilitation programmes can also be delivered through telemedicine with the assistance of the heart failure nurses and pharmacists in a comprehensive approach that includes structured telephone support, remote distance monitoring (e.g. weight, heart rate, blood pressure, etc.) and the use of implantable devices that can monitor and detect congestion.

Therefore, adverse outcomes in patients with heart failure can be prevented by using multimodal strategies that help the patient to reach clinical stability. These strategies are better served by a multiprofessional heart failure service.^62^

## Transitional of care interventions

Early rehospitalizations, in particular within 30 days after discharge, are an important performance measurement in some countries, such as the USA.^63^

However, several studies have shown that early rehospitalizations are poorly related to postdischarge mortality and an inverse relationship between 30-day rehospitalizations and mortality has even been shown.^64^

The vulnerability period poses an increased burden on patients and healthcare systems. It may also paradoxically represent a window of opportunity for clinicians, researchers and healthcare systems to develop appropriate and cost-effective strategies able to reduce the occurrence of negative outcomes. Indeed, those heart failure patients who are appropriately managed and overcome successfully this period tend to have a more benign prognosis and may experience a phase of long-term stability.

Transitional care interventions are mainly focussed on transition from hospital to home, and therefore from the acute setting to the immediate posthospitalization period and have the aim to further optimize guideline concordant medical therapy, build an appropriate management of comorbidities, recognize individual barriers to care, and identify reversible issues related to worsening heart failure, thus improving prognosis.^65^

According to the recommendations of the HFA of the ESC, a multidisciplinary approach is recommended for the management of heart failure patients in the vulnerable period.^18,62^ The aim of the multidisciplinary approach is to guarantee the continuity of care throughout the healthcare journey of heart failure patients, starting from the predischarge period and continuing after their discharge, with particular focus on the vulnerable period. Indeed, the discharge planning should start as soon as the patient's condition is stable (Fig. 1). The primary predischarge interventions included discharge planning, counselling and patient and caregiver health education.

A patient-centred approach and an active involvement of the patient and their family or carers in the plan of treatment, together with healthcare professionals, is a crucial factor in tailoring an individualized care plan to optimize the outcomes. Studies have shown that higher levels of social support from friends and family are associated with increased medication and dietary adherence; however, at the same time, it seems a variable associated with an increased risk for readmission.^66^

Key components to self-care are represented by periodic monitoring of heart failure symptoms and signs or activities, such as daily weighing, blood pressure and heart rate measurements; patient's adherence to medication and lifestyle changes; and possible active management, such as making changes to the dose of diuretic medication in response to a fluctuation in symptoms (Table 1).

**Table 1:** Patient monitoring

The heart failure multidisciplinary team (MDT) should work in collaboration with the primary care team, and should include:
A lead physician with training in heart failure (usually a consultant cardiologist) who is responsible for making the clinical diagnosis and directing the clinical management
A specialist heart failure nurse
A general practitioner
At discharge, the specialist heart failure MDT should prepare a summary that includes
Diagnosis, functional class and aetiology
Medicines prescribed, care plan for monitoring of medicines, indication on when medicines should be reviewed
Functional abilities and any social care needs
Follow-up care
Plan for a personalized, exercise-based cardiac rehabilitation programme
Monitoring of heart failure patients should include:
A full assessment of clinical status, functional capacity, fluid status, cardiac rhythm, cognitive status and nutritional status
A review of medications and up-titration, including need for changes and possible side effects
An assessment of renal function and electrolytes
The frequency of monitoring should depend on the clinical status and stability of the person
The monitoring interval should be short (up to 2 weeks) for patients discharged after an hospitalization for heart failure
If the clinical condition or medication has changed but is needed at least 6-monthly for stable people with proven heart failure
More detailed monitoring will be needed in patients with significant comorbidities or if their condition has deteriorated

Despite clear benefit, under-prescribing and a lack of guidelines for medical therapy optimization are still recognized problems across the continuum of care.

Recent observational studies comparing patients discharged on and off neurohormonal therapy indicate that up to 50% of early postdischarge mortality may be associated with guideline nonadherence.^67^ Several reasons can be attributed to the lack of adherence, such as polytherapy, not only side effects but also lack of understanding of discharge instructions, unaware of changes made to their medications or conflicting instructions received from the discharging physician and primary care physician.

Postdischarge strategies include general practitioner and specialist follow-up visits, telephone calls or home visits after discharge, facilitated access to care during periods of decompensation, and coordination of the tertiary healthcare structure wherever needed. Information should be individually tailored and take into account relevant comorbidities that may influence retention of information (such as cognitive impairment and depression), travel to the point of care (physical disability, living alone) or the need for psychosocial support for patients and their family/carers.^68^

Home telemonitoring can be useful in the postdischarge period, allowing easier communication, via phone or interactive internet-based interaction, between the patient and the healthcare providers to monitor healthy individuals or chronically ill patients remotely^69–71^ (Table 1).

Mobile health (mHealth) technologies have also emerged as a way to actively engage patients in their enrolment in the disease management programme and in the healthcare decision-making processes, through transmission of patient variables including blood pressure, weight and clinical symptoms, at regular time points. On the basis of these data, the provider can detect changes in patient status and initiate outpatient management changes early in the course so as to avoid hospitalizations.^72^

Technology can help in improving heart failure management through education programmes (such as heartfailurematters.org operated by the HFA of the ESC), and providing useful material for patients and their families.

Several smartphone applications (‘apps’) or devices to monitor physiological parameters are now available and can be used to record physical parameters and remind patients about the intake of medication to improve adherence. Lifestyle management programmes may be also useful in supporting rehabilitation. However, the transferability of data from home monitoring systems to their healthcare team is raising concerns regarding legal liability, data confidentiality and reimbursement, which is slowing the implementation of these technological changes in many countries.^28^

In the postdischarge phase, regular follow-up should be provided with the support of available technologies via telephone or home visits for chronically ill, high-risk or frail patients. Therefore, the key elements of this multidisciplinary programme include hospital heart failure physicians, cardiac rehabilitation units, specialized heart failure nurses, and a well structured network between primary care and tertiary centres.

The care plan of the postacute phase should start with discharge planning and it should commence as soon as the condition of the patient becomes stable and there are no signs of congestions. Information and education for self-care should be provided prior to discharge.^18^

Patients with heart failure benefit from regular follow-up and monitoring of biomedical parameters according to the recommendations of the ESC/HFA Guidelines on heart failure in order to ensure the safety and optimal dosing of medicines and detect the development of complications or disease progression. Monitoring may be undertaken by the patients themselves, during home visits by local heart failure nurses, community or hospital heart failure clinics and by remote monitoring. The optimal method and timing of monitoring will depend on local organizations and resources.

Ideally, patients should be assessed by their general practitioner within 1 week of discharge and the hospital cardiology team should follow up the patients within 2 and 4 weeks of discharge. Thereafter, follow-up visits should be planned according to patient status, need for medicines up-titration and need for further interventions.

The ideal timelines cannot be generalized as they have to be adapted to the local availability of services. Hospitals with early physician follow-up after discharge (typically within 7–14 days of heart failure discharge) show reduced 30-day readmission, and those that initiated programmes to discharge patients with an outpatient follow-up appointment already scheduled experienced a greater reduction in readmissions than those not taking up this strategy.^18^

Preventing unplanned short-term rehospitalization as a target for quality improvement can address unresolved acute illness, ongoing chronic illness and gaps in in-patient and outpatient care. Identifying patients at high risk of readmission can help not only to mitigate the clinical and financial burdens of patient care but also to allocate appropriate resources to these patients.

## Conclusion

The postacute phase of patients hospitalized because of decompensated heart failure is characterized by an increased risk of death and rehospitalization. A care plan should be developed for all patients prior to discharge and should include posthospital care, rehabilitation and implementation of medical therapy. All patients should receive the mainstay of treatment with ACEi/beta blockers/MRAs/SGLT2i as soon as possible; the up-titration of these medicines should be based on the clinical status, clinical signs and careful monitoring of heart rate, blood pressure, renal function and electrolytes. Renal function and iron status should be monitored and in iron-deficient patients intravenous iron should be used. Out-of-hospital management should be undertaken by heart failure nurses and general practitioners and can be facilitated by telemonitoring whenever possible. Cardiac rehabilitation should be recommended to all patients post discharge.

### Conflicts of interest

There are no conflicts of interest.
